# High expression of HSP60 and survivin predicts poor prognosis for oral squamous cell carcinoma patients

**DOI:** 10.1186/s12903-023-03311-5

**Published:** 2023-09-03

**Authors:** Ying Zhou, Yaoxiang Tang, Jiadi Luo, Yang Yang, Hongjing Zang, Jian Ma, Songqing Fan, Qiuyuan Wen

**Affiliations:** 1 Department of Pathology, The Second Xiangya Hospital, Central South University, Changsha, 410011 Hunan China; 2 Hunan Key Laboratory of Nonresolving Inflammation and Cancer, Cancer Research Institute of Central South University, Central South University, Changsha, 410011 Hunan China

**Keywords:** Oral squamous cell carcinoma, HSP60, Survivin, Prognosis, Biomarker

## Abstract

**Background:**

HSP60 is a heat shock proteins (HSPs) family member and help mitochondrial protein to fold correctly. Survivin is one of the inhibitors of apoptosis protein family member, which plays a significant part in cancer progression. They were capable of forming HSP60-survivin complexes and involved in the development of various tumors.

**Methods:**

The Cancer Genome Atlas (TCGA) database demonstrated that HSP60 and survivin and their correlation on mRNA expression level with OSCC patients. Besides, expression of HSP60 and survivin proteins was studied utilizing immunohistochemistry in tissue microarrays (TMA) in OSCC and in adjacent non-cancerous squamous epithelium (Non-CCSE) tissues.

**Results:**

Significantly increased levels of HSP60 and survivin in most cancers compared to normal tissue by pan-cancer analysis. HSP60 and survivin proved a significantly increased expression in OSCC samples compared to Non-CCSE both on mRNA and protein (both *P* < 0.05). Additionally, elevated HSP60 displayed a positive correlation with survivin in terms of mRNA and protein expression levels (all *P* < 0.001). Patients with OSCC who had advanced clinical stage or lymph node metastasis (LNM) showed higher HSP60 expression (*P* = 0.004, *P* = 0.006, respectively). Higher levels of the proteins HSP60 and survivin were significantly inversely correlated relationship with OSCC patients’ overall survival rates in multivariate survival analysis (*P* = 0.018, *P* = 0.040). From the above results, overexpression of HSP60 and survivin protein may serve as independent biomarkers predicting poor prognosis in OSCC.

**Conclusions:**

Elevated HSP60 and survivin might be served as novel poor prognosis biomarkers for surgically resected OSCC patients.

## Background

Oral cancer is a prevalent malignant tumor worldwide, with a high incidence and mortality rate, bringing a significant negative impact on global public health [1, 2]. Due to the late-stage diagnosis, extreme invasiveness, and therapy resistance, oral cancer had an utterly poor five-year survival rate of fewer than 50% [3]. Of all individuals with malignant oral cancer, accounting for more than 90% is oral squamous cell carcinoma (OSCC) [4]. The available treatments for OSCC patients, mainly chemotherapy, radiotherapy and surgery, are unable to capture the issues of drug resistance, recurrence and metastasis, leading to a poor prognosis [5]. The late diagnosis of OSCC patients leads to the high mortality; however, there aren’t many accurate biomarkers markers to predict tumor progression and patient prognosis to date [6]. Therefore, the in-depth study of OSCC molecular biomarkers is crucial to identify the malignancy of the disease, evaluate the prognosis and screen out novel therapeutic targets, all of these can contribute to more precise direction for OSCC cancer’s pathogenic mechanisms and targeted therapy.

Heat shock proteins (HSPs) known as a family of conserved mitochondrial chaperone with critical roles in maintaining cellular homeostasis in response to hyperthermia, chemical agents, and other stress conditions [7]. Heat shock protein 60 (HSP60) as one of these mitochondrial molecular chaperones, interacts with other proteins, including p53, survivin, the inhibitor of NF-B (IKK), and others, to control tumor metabolism, metastasis, apoptosis, and treatment tolerance [8, 9]. HSP60 was shown to be overexpressed in several tumor types, which include non-small cell lung cancer (NSCLC) [10], colorectal cancer [11], breast cancer [12] as well as ovarian cancer [13] and HSP60 overexpression has also been linked with poor prognosis. In light of this, HSP60 has been suggested as a biomarker for the development and progression of tumors in cancer, which has sped up the development of HSP60 inhibitors for targeted therapy.

As an anti-apoptosis protein belongs to apoptosis protein (IAP) family, survivin contains a baculovirus IAP repeats (BIR) domain specific to the formation of dimer and the inhibition of apoptosis [14], to play key roles in controlling cellular division and inhibiting apoptosis by blocking caspase activation [15]. Studies have demonstrated almost no survivin expression detected in differentiated normal tissues but highly and selectively expressed in almost all human malignancies [16]. Therefore, it is reasonable to conclude that survivin takes a significant part in cancer progression and proposed as a potential cancer therapeutic target.

Currently, inhibition of tumor cell apoptosis is a novel biomarker of the occurrence and development of most or all multiple types of tumors. Nevertheless, the precise relationship between HSP60 and survivin in OSCC is currently unknown. Therefore, in our current investigation, 79 patients of OSCC and 22 cases of Non-CCSE had HSP60 and survivin protein detected by IHC in tissue microarrays (TMA). Thus, this study aimed to detect the expression of HSP60 and survivin and thereby explore the association between the expression of those two proteins and clinicopathological features in OSCC.

## Methods

### TCGA database

We collected the TCGA database to conduct a pan-cancer bioinformatics analysis of HSP60 and BIRC5. And we used 32 (normal control, NC) and 329 OSCC samples from TCGA database to make tumor/normal differential expression of HSP60 and survivin and analyze their correlation.

### Ethical statement

The Ethics Committee of the Second Xiangya Hospital of Central South University approved this study. All experimental procedures were in accordance with the 1964 Helsinki declaration and its later amendments of comparable ethical standards. Written informed consent was obtained from every research sample. For the minors, however, the guardian must sign a written consent on their behalf.

### Clinical data and TMA

In this study, all OSCC patient with OSCC were submitted to surgical treatment at the Department of Oral and Maxillofacial Surgery, the Second Xiangya Hospital of Central South University (Changsha, China) from 2007 to 2011. All OSCC samples and non-cancerous squamous epithelium were obtained from Department of Pathology, the Second Xiangya Hospital of Central South University. These patients had undergone standard staging, a decisive surgical removal of a portion of the tongue, and a methodical neck lymph node dissection. According to the WHO’s histological categorization of oral cancer, OSCC was confirmed histologically in all patients. In accordance with the 8th edition of the AJCC/UICC TNM staging system of OSCC, the current analysis’ staging classification was completed. No patients had been previously treated with chemotherapy and radiotherapy at the time of original operation. All patients’ full clinical histories and follow-up information were accessible. Informed consent was obtained from all patients before surgery. Follow-up time for analyses of survival for the 79 OSCC cases was calculated from the date of surgery to the date of death, loss-to-follow-up, or 2011, whichever came first. The characteristics of patients were detailed in Table 1. Tissue microarrays containing two tumor cores and two Non-CCSE cores in Non-CCSE in TMAs were created using TMA technology with the previously described procedure [17]. The perforation diameter of each sample was performed 0.6 mm on average.

**Table 1:** 79 cases of oral squamous cell carcinoma (OSCC) patients feature

Patients’ characteristics	No. of patients (%)
**Age(years)**	
< 50	29(36.7)
≥ 50	50(63.3)
**Gender**	
Male	64(81.0)
Female	15(19.0)
**Cigarette**	
Yes	48(60.8)
No	31(39.2)
**Alcohol**	
Yes	41(51.9)
No	38(48.1)
**Areca nut**	
Yes	34(43.0)
No	45(57.0)
**Differentiation**	
Well	58(73.4)
Moderate	13(16.5)
Poor	8(10.1)
**Clinical stages**	
Stage I	23(29.1)
Stage II	19(24.1)
Stage II	17(21.5)
Stage IV	20(25.3)
**Lymph node status**	
N0	51(64.6)
N1/N2/N3	28(35.4)
**Survival status**	
Alive	56(70.9)
Death	23(29.1)

### Antibody selections

The following primary antibodies were used for staining: HSP60 (Catalog: #12,165, Cell Signaling Technology; 1:4000 dilution); survivin (Catalog: #2808, Cell Signaling Technology; 1:2000 dilution) at 4 °C overnight.

### IHC and scores

IHC staining of HSP60 and survivin in OSCC and Non-CCSE TMAs was performed using the previous protocol [18]. As a negative control, matched IgG isotype antibody was used to confirm the specificity of the antibody. Each experiment had a positive control slide.

HSP60 and survivin staining in TMAs were evaluated at 200x magnification using light microscopy by two pathologists (SF and QW) blinded to the clinicopathologic data of the patients. A formula to calculate the overall expression marks based on the intensity and distribution of IHC staining was created: total score = intensity mark + percentage mark. Simply put, we visually regard as 0 (negative), 1 (weak), 2 (moderate), or 3 (strong) to rate the intensity of staining for HSP60 and survivin. Meanwhile, positive tumor cell percentages were assigned as 0 (0%), 1 (1–25%), 2 (26–50%), 3 (51–75%), and 4 (76–100%). All samples had a total score between 0 and 7 for HSP60 and survivin protein levels. Also, on the basis of overall survival (OS) of OSCC using the log-rank test, the optimal critical levels of the two proteins were 5 and 4, respectively. A score above 5 and 4 indicated high expression of the two proteins, whereas lower scores indicated poor expression in final analysis. Two pathologists reached consensus on all IHC scoring disagreements, and their level of agreement was 95%.

### Statistical analysis

SPSS version 24.0 software was utilized to conduct statistical analysis. The correlation between HSP60 and survivin, as well as analysis of survival rate curve and survival rate, were evaluated through the use of the log-rank test, Kaplan-Meier analysis, the Spearman’s rank correlation coefficient and regression analysis, respectively. The diagnosis to date of death was utilized to compute overall survival rates. Cox proportional hazard regression model was carried out to evaluate if HSP60 and survivin are independent prognostic markers. Every P value was derived from a statistical analysis with two branches, and a value of p < 0.05 was used as the cutoff for determining whether or not the results were statistically significant.

## Results

### Bioinformatics analysis of HSP60 and survivin in pan-cancer

**Fig. 1:**
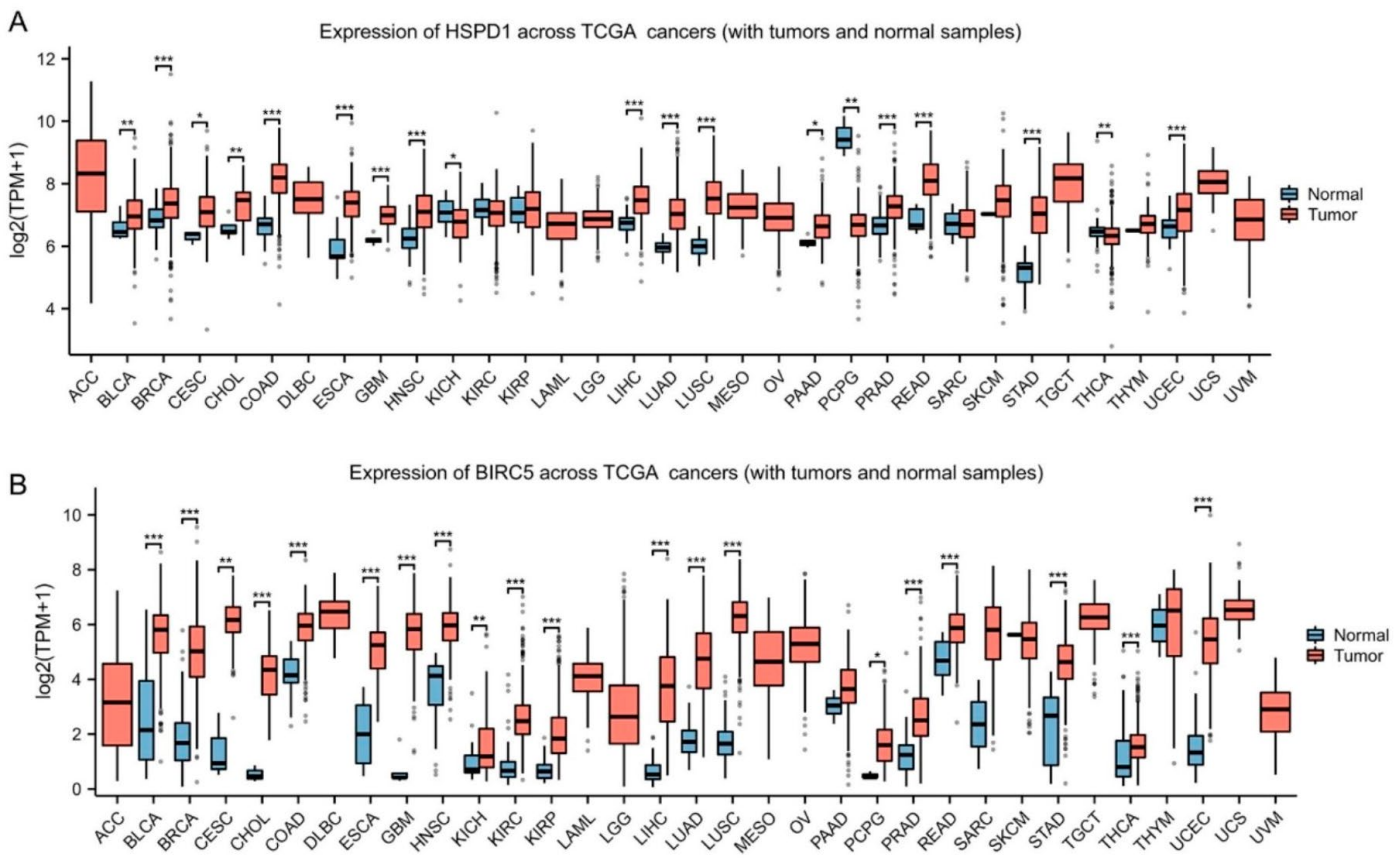
Analysis of HSP60 and survivin expression in pan-cancer Comparison of mRNA expression of HSP60 (**A**) and survivin (**B**) between tumor and normal tissues. Both HSP60 and survivin are upregulated in most malignant tumors. Adrenocortical carcinoma (ACC); Bladder Urothelial Carcinoma (BLCA); Breast invasive carcinoma (BRCA); Cervical squamous cell carcinoma and endocervical adenocarcinoma (CESC); Cholangio carcinoma (CHOL); Colon adenocarcinoma (COAD); Lymphoid Neoplasm Diffuse Large B-cell Lymphoma (DLBC); Esophageal carcinoma (ESCA); Glioblastoma multiforme (GBM); Head and Neck squamous cell carcinoma (HNSC); Kidney Chromophobe (KICH); Kidney renal clear cell carcinoma (KIRC); Kidney renal papillary cell carcinoma(KIRP); Acute Myeloid Leukemia (LAML); Brain Lower Grade Glioma (LGG); Liver hepatocellular carcinoma (LIHC); Lung adenocarcinoma (LUAD); Lung squamous cell carcinoma (LUSC); malignant mesothelioma (MESO); Ovarian serous cystadenocarcinoma (OV); Pancreatic adenocarcinoma (PAAD); Pheochromocytoma and Paraganglioma (PCPG); Prostate adenocarcinoma (PRAD); Rectum adenocarcinoma (READ) ; Sarcoma (SARC); Skin Cutaneous Melanoma (SKCM); Stomach adenocarcinoma (STAD); Testicular Germ Cell Tumors (TGCT); Thyroid carcinoma(THCA); Thymoma (THYM); Uterine Corpus Endometrial Carcinoma (UCEC); Uterine Carcinosarcoma (UCS); Uveal Melanoma (UVM).

Significantly increased levels of HSP60 (Fig. 1A) and survivin (Fig. 1B) in most cancer compared to normal tissue by pan-cancer analysis using TCGA data. As shown in Fig. 1A, it showed that there were higher HSPD1 levels in 19 cancer samples compared to normal samples (Fig. 1A), including breast invasive carcinoma (BRCA), colon adenocarcinoma (COAD), esophageal carcinoma (ESCA), head and neck squamous cell carcinoma (HNSC), rectum adenocarcinoma (READ) and stomach adenocarcinoma (STAD). And compared with normal tissues, BIRC5 expression is significantly higher in tumor tissues samples from patients with 20 out of 33 tumor types, including bladder urothelial carcinoma (BLCA), BRCA, HNSC, lung adenocarcinoma(LUAD), lung squamous cell carcinoma (LUSC) and READ (Fig. 1B). These results suggested that HSPD1 and BIRC5 expression levels may be associated with tumorigenesis in most types of human cancer.

### Bioinformatics analysis of HSP60 and survivin in OSCC

**Fig. 2:**
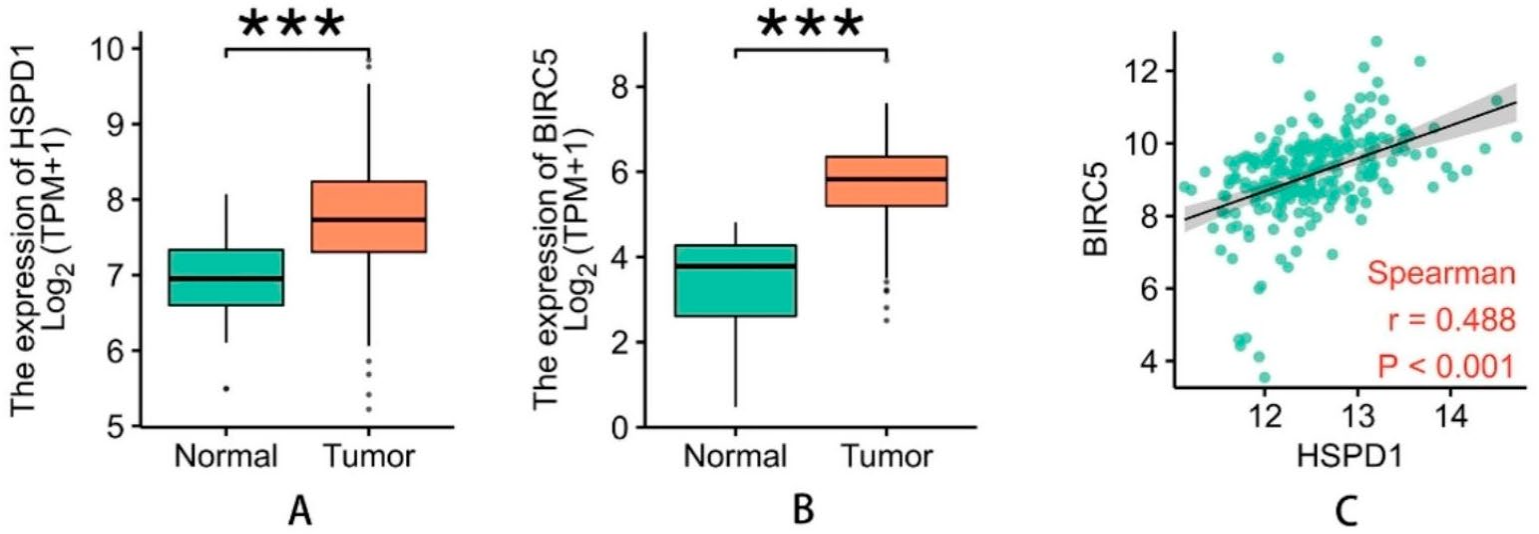
Bioinformatics analysis of HSP60 and survivin in OSCC mRNA expression of HSP60 (**A**) and survivin (**B**) was significantly higher in OSCC tissues than that in NC (normal control) tissues (Both *P* < 0.0001). HSP60 was positively correlated with survivin in mRNA level (**C**) (r = 0.488, *P* < 0.001)

We selected a total of 329 OSCC tissues and 32 non-cancerous tissue samples based on the TCGA database. The results showed that when compared to NC tissues, the expression of HSP60 mRNA was considerably higher in OSCC tissues (*P* < 0.05). In line with this finding, the expression of HSP60 was found to have a positive correlation with survivin mRNA levels in patient tumors (r = 0.488, *P* < 0.0001; Fig. 2).

### Elevated expression of HSP60 and survivin proteins was evidently higher in OSCC

**Fig. 3:**
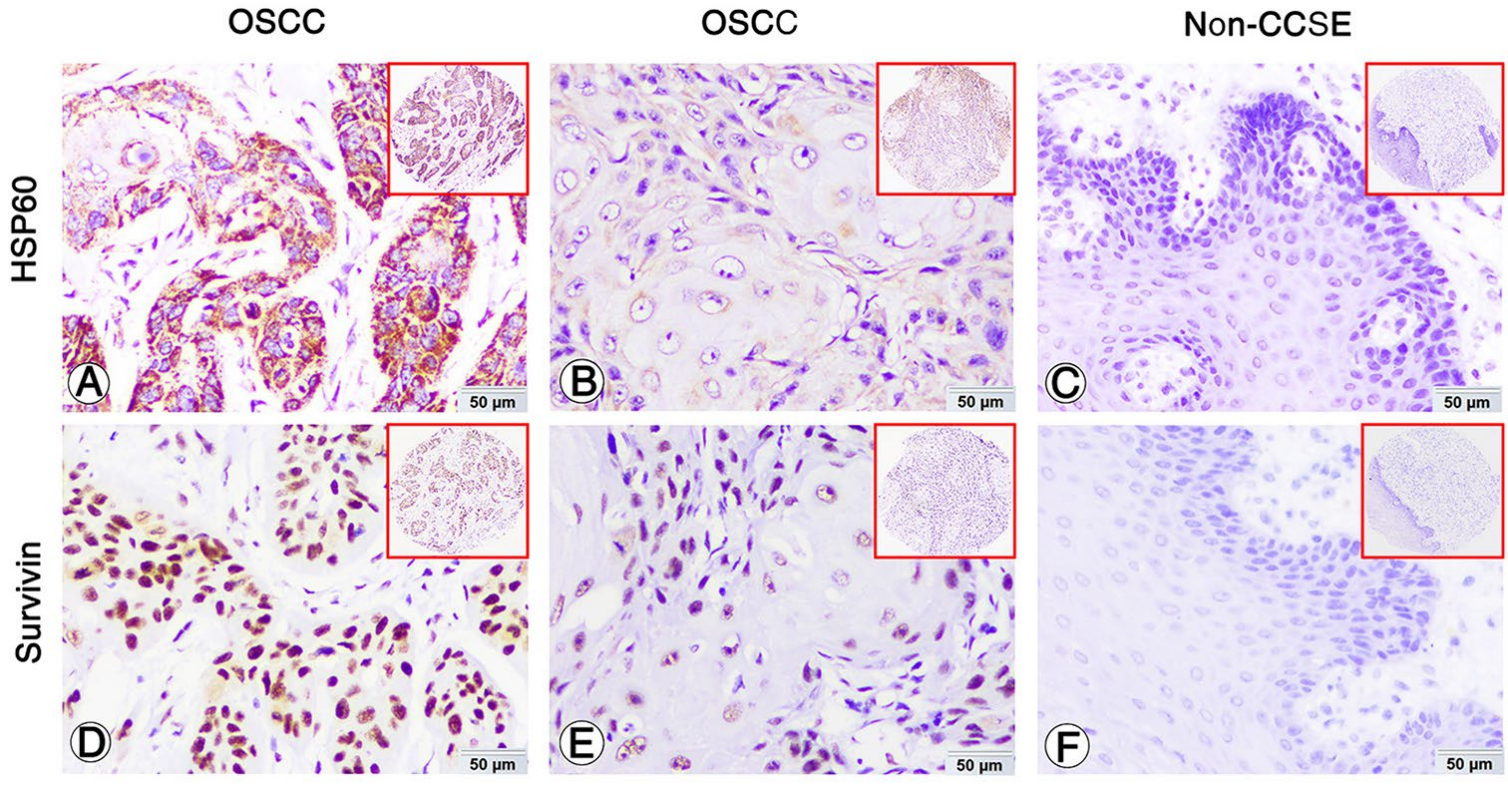
Expression of HSP60 and survivin in OSCC and Non-CCSE were detected by IHC Expression of HSP60 and survivin in OSCC and Non-CCSE tissues by IHC. Strong positive cytoplasm staining and weak staining of HSP60 in OSCC patients (**A** and **D**). Strong positive nuclear staining and weak staining of survivin in OSCC patients (**B** and **E**). Negative staining of HSP60 (**C**) and survivin (**F**) proteins was found in Non-CCSE.

**Fig. 4:**
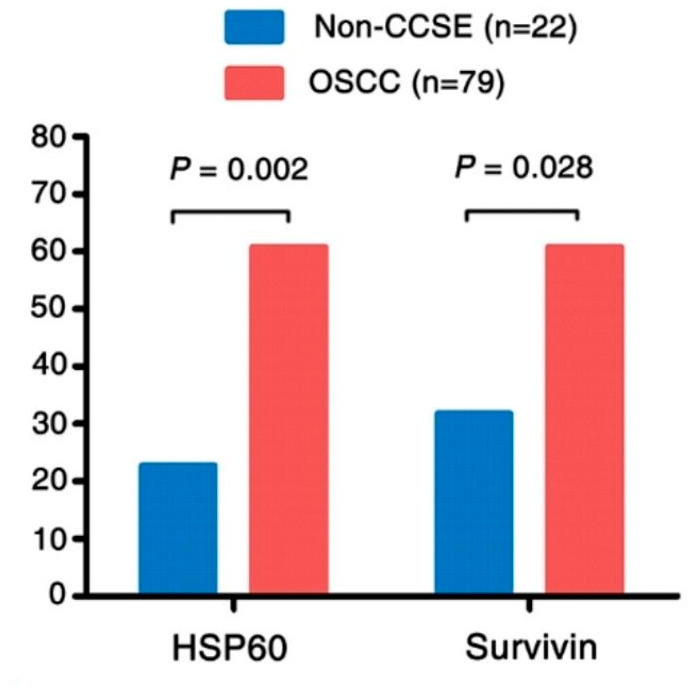
The comparison of expression of HSP60 and survivin in OSCC compared to the Non-CCSE The expression of HSP60 and survivin in OSCC was significantly higher than those in Non-CCSE (*P* < 0.002, *P* = 0.028, respectively)

IHC was performed to examine positive expression and subcellular location of HSP60 and survivin proteins in OSCC and the Non-CCSE tissues. The positive staining of HSP60 and survivin was observed in the cytoplasm and nuclear (Fig. 3A and D, B and E, respectively). The staining of the two proteins is negative in Non-CCSE tissues (Fig. 3C and F). The proportion of high HSP60 was 60.8% (48/79) and survivin protein showed the same high expression percentage in OSCC. The data in Non-CCSE were 22.7% (5/22) and 31.8% (7/22) respectively. HSP60 and survivin proteins in OSCC tissues were markedly upregulated as shown in Fig. 4.

### Relationship between clinicopathological features and high expression of HSP60 and survivin proteins in OSCC

We then used the univariate chi-square test to examine the relationships between HSP60 and survivin expression and a variety of clinicopathological parameters of OSCC patients, including gender, age, clinical stages, lymph node metastasis (LNM) status, pathological differentiation, and living habits like tobacco use, alcohol consumption, and areca nut chewing. As observed in Table 2, high expression of HSP60 protein correlated favorably with clinical stage (*P* = 0.006), although expression of survivin was not significantly correlated with clinical stage (*P* > 0.05). Higher levels of HSP60 expression were found in patients with LNM status in OSCC (*P* = 0.004). The data presented in Table 2 suggested that a significant positive association existed between the elevated expression of survivin and the pathological differentiation of OSCC patients (*P* = 0.047). Age, gender, smoking, drinking, and chewing areca nuts were not found to have any prognostic significance in OSCC (all *P* > 0.05).

**Table 2:** Analysis of the association between expression of HSP60 and survivin protein and clinicopathological features of oral squamous cell carcinoma (OSCC) (n = 79)

Clinicopathological	HSP60			Survivin		
features (n)	High (%)	Low (%)	*P*	High (%)	Low (%)	*P*
**Age**						
< 50 years(n = 29)	14(48.3)	15(51.7)		15(51.7)	14(48.3)	
≥ 50 years(n = 50)	34(68.0)	16(48.4)	0.084	33(66.0)	17(34.0)	0.239
**Gender**						
Male(n = 64)	39(60.9)	25(39.1)		39(60.9)	25(39.1)	
Female(n = 15)	9(60.0)	6(40.0)	1.000	9(60.0)	6(40.0)	1.000
**Cigarette**						
Yes(n = 48)	31(64.6)	17(35.4)		29(60.4)	19(39.6)	
No(n = 31)	17(54.8)	14(45.2)	0.480	19(61.3)	12(38.7)	1.000
**Alcohol**						
Yes(n = 41)	25(61.0)	16(39.0)		24(58.5)	17(41.5)	
No(n = 38)	23(60.5)	15(39.5)	1.000	24(63.2)	14(36.8)	0.818
**Areca nut**						
Yes(n = 34)	18(52.9)	16(47.1)		23(67.6)	11(32.4)	
No(n = 45)	30(66.7)	15(33.3)	0.250	25(55.6)	20(44.4)	0.354
**Differentiation**						
Well(n = 58)	33(56.9)	25(43.1)		38(65.5)	20(34.5)	
Moderate(n = 13)	10(76.9)	3(23.1)		4(30.8)	9(69.2)	
Poor(n = 8)	5(62.5)	3(37.5)	0.407	6(75.0)	2(25.0)	0.047*
**Clinical stages**						
**Stage** I−II (n = 43)	20(46.5)	23(53.5)		27(62.8)	16(37.2)	
**Stage** III−IV (n = 36)	27(77.8)	9(22.2)	0.006*	21(58.3)	15(41.7)	0.818
**LNM**						
LNM (n = 28)	23(82.1)	5(17.9)		16(57.1)	12(42.9)	
No LNM (n = 51)	37(49.0)	13(51.0)	0.004*	32(62.7)	19(37.3)	0.639
**Survival status**						
Alive (n = 56)	29(51.8)	27(48.2)		35(62.5)	21(37.5)	
Death (n = 23)	19(82.6)	4(17.4)	0.012*	13(56.5)	10(43.5)	0.623

*Chi-square test, statistically significant difference (*P* < 0.05)

Abbreviations: LNM, lymph node metastasis

### The correlation between HSP60 and survivin expression in OSCC

We examined the relationship between HSP60 and survivin expression in OSCC using these data. Table 3 shows that in patients with OSCC, increased HSP60 expression was positively correlated with survivin expression (r = 0.363, *P* < 0.001; R^2^ = 0.2119, *P* < 0.0001, respectively).

**Table 3:** The pairwise association between expression of HSP60 and Survivin in OSCC.

	HSP60	
**Survivin**	Spearman’s	Regression
r	r = 0.363	R^2^ = 0.2119
Sig. (2-tailed)	0.001*	< 0.0001

*Spearman’s rank correlation test and regression analysis, statistically significant difference. * *P* < 0.05

### Effects of high HSP60 and survivin expression on patients’ prognosis

We utilized Kaplan-Meier survival curves of all 79 OSCC patients, and the statistical significance was evaluated by the log-rank test when examining the effect of HSP60 and survivin protein expression on OSCC patient survival. There were 23 (29.1%) total deaths among OSCC patients at the time of the study.

**Fig. 5:**
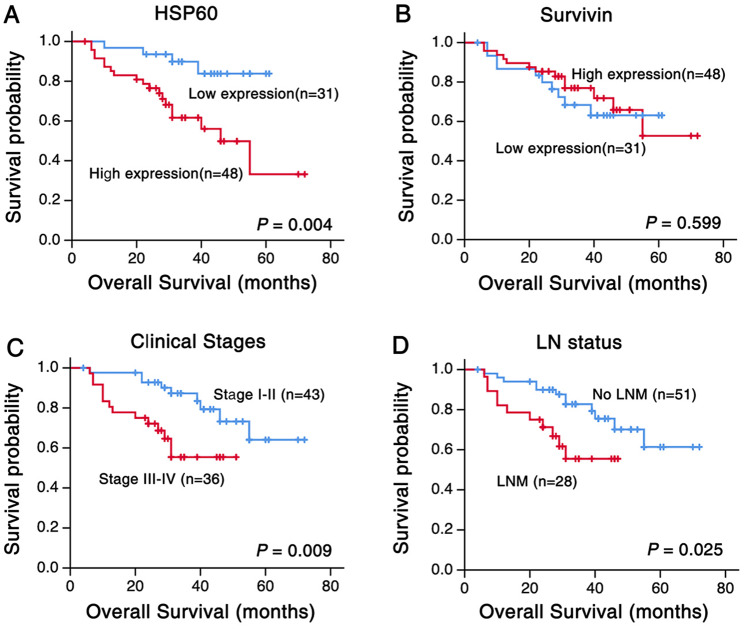
Kaplan-Meier cures for overall survival of OSCC patients with expression of HSP60 and survivin (**A**) OSCC patients with high expression of HSP60 showed worse overall survival rates compared to patients with low HSP60 expression (*P* = 0.004, two sided). (**B**) There was no statistical significance of overall survival rate between OSCC patients with different phenotype of survivin protein (*P* = 0.599, two sided). (**C**) Overall survival rate of OSCC patients with clinical stage I-II is higher than that of patients with clinical stage III-IV (*P* = 0.009, two sided). (**D**) Overall survival rate of OSCC patients with lymph node metastasis was significantly lower than that of patients without lymph node metastasis. (*P* = 0.025, two sided)

Figure 5 depicted the Kaplan-Meier survival plots for OSCC patients with different expression of HSP60 and survivin protein (Fig. 5). Univariate survival (log-rank test) analysis showed that the overall survival rates for OSCC patients with low HSP60 expression were significantly higher than these with high HSP60 expression (Fig. 5A, P = 0.004). However, no prognostic significance was noticed between the survivin expression and patients’ overall survival rate (Fig. 5B, P = 0.599). We made the survival curves of OSCC patients based on lymph node status and clinical stages. Figure 5C and D show that OSCC survival rates improved when diagnosed at an early stage (stages I and II) or when LNM was absent. Overall survival was shorter for patients with stage III or IV OSCC and LNM compared to stage I or II OSCC and without LNM (*P* = 0.009, Fig. 5C, and *P* = 0.025, Fig. 5D, respectively).

Multivariate Cox proportional hazard regression analysis was used to further verify whether the expression of HSP60 and the survivin protein were the independent prognostic factors in OSCC patients, which was show in Table 4. In the course of performing a multivariate analysis on the expression of HSP60 and survivin in 79 OSCC patients, which included clinical stages, LNM status, pathological differentiation, tobacco using, alcohol consumption and areca nuts chewing. According to what we have discovered, the presence of high levels of HSP60 and survivin protein was proved to be independent poor factors for OSCC (*P* = 0.018, *P* = 0.040, respectively), as well as tobacco using (*P* = 0.009). Besides, no impact was detected with alcohol, pathological differentiation, clinical stages, LNM status of OSCC (all *P* > 0.05).

**Table 4:** Summary of multivariate analysis of Cox proportional hazard regression for overall survival in 79 cases of OSCC.

Parameter	B	S.E.	Wald	*P*	Exp(B)	95.0%CI for Exp(B)	
						Lower	Upper
**HSP60 expression**	1.600	0.676	5.604	0.018*	4.954	1.317	18.633
**Survivin expression**	-1.074	0.524	4.208	0.040*	0.342	0.122	0.953
**Cigarette**	1.972	0.751	6.893	0.009*	7.183	1.648	31.302
**Areca nut**	-1.223	0.564	4.701	0.030*	0.294	0.097	0.889
**Alcohol**	-1.141	0.620	3.381	0.066	0.320	0.095	1.078
**Differentiation**	0.163	0.790	0.043	0.836	1.177	0.250	5.535
**Clinical stages**	1.164	0.739	2.485	0.115	3.204	0.753	13.626
**LNM status**	-0.716	0.731	0.959	0.328	0.489	0.116	2.049

Abbreviations: LNM, lymph node metastasis; CI, confidence interval

Note: multivariate analysis of Cox regression, *P<0.05

## Discussion

We came to the conclusion that the levels of HSP60 and survivin expression in OSCC were significantly higher in mRNA as well as protein when compared to adjacent Non-CCSE tissues in current research. HSP60 expression was found to be higher in patients with advanced clinical stages and LNM and demonstrated a strong connection between high survivin expression and poor pathological differentiation of OSCC patients. Additionally, those with OSCC who had increased HSP60 expression exhibited a poorer survival rate than those with decreased HSP60 expression. These statistics indicated that overexpression of HSP60 and survivin has potential prognostic value and could be new prognostic marker for OSCC. HSP60 as one of the most common molecular chaperones is able to support cancer cell survival under adverse physiological and stress circumstances like hypoxia, viral agents, low pH as well as exposure to UV light and chemical [19]. HSP60 expression associates with cancer cell survival and apoptosis by regulating caspase activation and with cancer metastasis through interacting with β-catenin [20]. The aberrant HSP60 intimately associated with the occurrence and progression in various types of malignancies, including pancreatic cancer [21], hepatocellular carcinoma [22], and esophageal squamous cell carcinoma [23], and in regards to the poor prognosis of patients. Our data revealed that overexpression of HSP60 and survivin protein could act as independent prognostic indicator of OSCC.

HSP60 staining was observed in the cytoplasm in our present study, which is consistent with the previously founding in patients with colorectal cancer [11]. Furthermore, HSP60 protein was only weakly expressed positively or negatively in the squamous epithelial layer of non-cancerous squamous control tissues, whereas positive HSP60 expression was markedly elevated in OSCC compared to adjacent Non-CCSE control tissue. Hence, HSP60 may be crucial in fostering the progression and development of OSCC, and targeted inhibition of HSP60 is a safe anti-cancer strategy. Targeted or combination therapy based on HSP60 inhibitors may be a promising avenue for enhancing the efficacy of cancer treatment since HSP60 inhibitors impact the molecular chaperone activity or post-translational modification of HSP60 [8]. Survivin has drawn attention as a distinct member of the IAP gene family with a potential dual role in inhibiting apoptosis and regulating mitosis and is widely regarded as potential therapeutic target to date [24, 25]. In cell lines and animal models, it has been reported that over-expressing survivin inhibits both the intrinsic and extrinsic apoptosis pathways, which has been linked to a rise in resistance to cancer treatments [26]. Survivin serves as both a survival mechanism and an inhibitor of apoptosis, making it a prime target for combating drug resistance [27]. It offers a chance to overcome resistance by incorporating survivin inhibitors into combination chemotherapy, such as prostate cancer cells can be effectively treated with 7F1 and docetaxel combined with survivin dimerization inhibitors to overcome docetaxel resistance [26]. However, more research is needed to determine whether survivin inhibitors are also effective against OSCC or whether they work in concert with chemotherapy to treat these cancers.

Both the mRNA and protein levels of HSP60 and survivin showed a positive correlation in the data, and the presence of elevated HSP60 and survivin expression revealed that these parameters were poor independent prognostic factors for OSCC patients’ survival. It has been proved that HSP60 helps to stabilize mitochondrial survivin and knockdowns of mitochondrial HSP60 can promote apoptosis though breaking the interaction of HSP60-survivin in tumor cells [28, 29]. Furthermore, HSP60 has the ability to interact with survivin resulting in the formation of HSP60–survivin complexes to stabilize survivin, which in turn promotes cancer cell survival in a variety of cancer cell types [21, 30].

The use of tobacco and heavy alcohol drinking are the two most important risk factors for OSCC [31], and other known risk factors including areca nut, narcotics, epigenetic factors and viral infections [32, 33]. In this study, multivariate analysis proved that tobacco was an independent factor for poor prognosis in OSCC patients and there was no statistically significant difference for alcohol and OS. Nonetheless, according to previous study of our research team [34], similar result that areca nut showed as a protective factor of OSCC were found in our present study. Extensive epidemiologic evidence has demonstrated that consumption of areca nut is a prevailing risk for OSCC with more than 400 million people habitually eat areca nuts in Southeast Asian and other countries and regions, including Hunan Province of China (especially the Xiangtan City) [35–37]. On the one hand, considering the regional nature of areca nut chewing, it is possible that the contradiction observed could be attributed to sampling errors within the research samples. On the other hand, some of our patients only occasionally or irregularly chew betel nuts, however, we did not separate them from those who chew betel nuts for a long time.

However, the specific mechanisms and other players involved in the relationship between HSP60 and survivin have not been characterized. Our work on the creation of HSP60 and survivin inhibitors may be appealing, offering a promising line of inquiry for targeted cancer therapy and reduce both the adverse effects and drug resistance while also enhancing the efficiency of targeting cancerous cells.

## Conclusions

Summing up our study, OSCC patients had clearly elevated HSP60 and survivin protein expression. Overexpression of HSP60 and survivin could be viewed as novel biomarkers for poor prognosis, who possess potential application value for OSCC targeted therapy.

## Data Availability

The datasets used and/or analyzed during the current study are available from the corresponding author on reasonable request.
